# Thoracoscopic esophagectomy in total pharyngolaryngoesophagectomy for esophageal cancer; A case series

**DOI:** 10.1016/j.amsu.2020.10.006

**Published:** 2020-10-12

**Authors:** Yasue Kimura, Hiroshi Saeki, Qingjiang Hu, Yuichi Hisamatsu, Mioko Matsuo, Sei Yoshida, Eiji Oki, Ryuji Yasumatsu, Hideaki Kadota, Masaki Mori

**Affiliations:** a Department of Surgery and Science, Graduate School of Medical Sciences, Kyushu University, Japan; b Department of General Surgical Science, Graduate School of Medicine, Gunma University, Japan; c Department of Otorhinolaryngology, Graduate School of Medical Sciences, Kyushu University, Japan; d Department of Plastic Surgery, Graduate School of Medical Sciences, Kyushu University, Japan

**Keywords:** Esophageal cancer, Total pharyngolaryngoesophagectomy (TPLE), Thoracoscopic esophagectomy

## Abstract

**Background:**

Total pharyngolaryngoesophagectomy (TPLE) is associated with major complications and is extremely invasive. In 2011, our institution introduced thoracoscopic esophagectomy in the left hemi-prone position and laparoscopic reconstruction with a gastric tube in patients undergoing TPLE. Herein, we describe the use of this operative method in 26 patients, focusing on the technical aspects of the surgery.

**Materials and methods:**

From January 2011 to December 2018, 26 patients underwent minimally invasive TPLE with gastric tube reconstruction in our institute. The thoracoscopic procedure was performed with the patient in the semi-prone position. The patient was then moved to the supine position, and the laparoscopic procedure and pharyngolaryngectomy were started simultaneously. After pharyngolaryngectomy, microvascular anastomoses or free jejunal flap interposition were performed at the site of the gastric tube reconstruction. The data from these 26 patients were retrospectively analyzed.

**Results:**

The median age was 66 years, and 3.8% of the patients were female. The Union for International Cancer Control stages of esophageal cancer were 0 (*n* = 2), I (*n* = 4), II (*n* = 7), III (*n* = 8), and IV (*n* = 5). Eight patients had concomitant esophageal cancer and head and neck cancer. Reconstruction with only a narrow gastric tube was used in 16 patients, while free jejunal flap interposition was used in 10 patients. The surgical procedures resulted in minimal complications. Postoperative complications of Clavien-Dindo grade ≥1 included anastomotic leakage in two patients and pneumonia in one.

**Conclusion:**

Thoracoscopic esophagectomy in the left hemi-prone position and laparoscopic reconstruction with a gastric tube in patients undergoing TPLE was safe and effective. The complications were improved via the development of various procedures. Further improvement is necessary before this thoracoscopic approach is established as a standard procedure for TPLE.

## Highlights

• Total pharyngolaryngoesophagectomy (TPLE) is invasive and complicated procedure.
• The frequency of complications of thoracoscopic TPLE are less than that of thoracotomy.
• Minimally invasive thoracoscopic TPLE for esophageal cancer is safe and feasible.

## 1 Introduction

Carcinoma of the hypopharynx is associated with poor survival, largely because tumors in this region remain asymptomatic until the disease reaches an advanced stage [1,2]. Stage III or IV hypopharyngeal cancer often invades the cervical esophagus, which is a very aggressive cancer with a poor prognosis. Pharyngolaryngeal and thoracic esophageal cancer also frequently occur concomitantly [3], leading to a high mortality rate. The standard treatment for these cancers is surgical resection; however, this surgery is extremely invasive, as it frequently requires both the thoracic and abdominal approaches [4].

Total pharyngolaryngoesophagectomy (TPLE) is mainly indicated either for synchronous cancer of the thoracic esophagus and the head and neck or for cervical-thoracic esophageal cancer. TPLE is considered to be the most complicated and most invasive gastrointestinal tract surgery due to the unstable blood flow of the reconstructed conduit and the extremely wide resection field. To overcome these problems, the application of techniques such as staged operations, muscular flaps, and microvascular anastomosis [5,6].

Esophageal malignancies are increasingly being treated by minimally invasive esophagectomy (MIE) with lymph node dissection in the prone position, without thoracotomy and laparotomy, in an attempt to reduce the mortality and morbidities, such as pulmonary complications [7]. MIE was introduced in our institution in 2010, and has been applied in TPLE since 2011. The safety of minimally invasive TPLE comprising both thoracoscopic and laparoscopic surgery, and the plastic surgery technique [8]. As there are still few facilities in the world that routinely perform complicated TPLE with minimally invasive thoracoscopic and laparoscopic procedures, we herein report our experience in performing such TLPE in a case series of 26 patients.

## 2 Patients and methods

### 2.1 Patient selection

A retrospective review of our institutional records revealed that 26 (8.5%) of 305 patients who underwent surgery for esophageal cancer from January 2011 to December 2018 received TPLE with MIE. This work has been reported in line with the PROCESS criteria [9].

### 2.2 Surgical procedure

#### 2.2.1 Minimally invasive esophagectomy

Esophagectomy was performed by a board-certified esophageal surgeon [10]. Surgery was started with the patient under general anesthesia in the left semi-prone position. Two 12 mm ports and two 5 mm ports were inserted into the intrathoracic space, and artificial CO_2_ pneumothorax was achieved at a pressure of 7–10 mmHg. With the patient in the left semi-prone position, the trachea must be moved to enable the surgeon to access the left upper mediastinal lymph nodes for dissection. A rigid endotracheal tube, such as a Carlens tube, was not used to selectively ventilate each lung, as such tubes make it difficult to move the trachea to access the left upper mediastinal lymph nodes. In our experience, artificial pneumothorax alone did not achieve adequate desufflation of the right lung; therefore, since 2019, we have used both a bronchial blocker and artificial pneumothorax to obtain sufficient desufflation for lymph node dissection. Esophagectomy and lymph node dissection were performed. During this procedure, the bronchial artery was preserved to prevent bronchial necrosis.

#### 2.2.2 Laparoscopic dissection of abdominal lymph nodes and creation of a gastric conduit

The patient was moved to the supine position, and the laparoscopic procedure and pharyngolaryngectomy were started simultaneously. The laparoscopic procedure was performed via five ports. The greater curvature of the stomach was dissected, the right gastroepiploic vessels were preserved, and the left gastric vessels were ligated, clipped, and cut. The lesser curvature and abdominal lymph nodes were dissected. The esophageal hiatus was dissected, and the lower esophagus was pulled out from the mediastinum. The esophagogastric organ was removed from the peritoneal cavity through an umbilical skin incision, and a 3.0–3.5-cm-wide gastric tube was created using a linear stapler. The blood flow of the gastric tube was checked using the indocyanine green fluorescence angiography method [11].

#### 2.2.3 Pharyngolaryngectomy

A U-shaped skin incision was made in the cervical site. The larynx and hypopharynx were raised, and the cervical esophagus was released from the trachea. The trachea was cut just above the clavicle, and the pharynx was cut. Cervical lymph node dissection was performed, and the specimen was excised.

#### 2.2.4 Reconstruction and microvascular anastomosis

The gastric tube was inserted into the right thoracic cavity through the esophageal hiatus and pulled up to the neck through the mediastinal route.

In cases where the gastric tube was not long enough to reach the anastomotic site of the pharynx, a free jejunal flap (FJF) was interposed. Laparoscopy revealed the Treitz ligament and the orientation of the proximal jejunum. A 20–40-cm-long section of the jejunum including the second branch of the jejunal artery was dissected from the Treitz ligament as the FJF for reconstruction. The FJF was anastomosed with the stump of the hypopharynx on the oral side in an end-to-end manner using 3–0 PDS. The anal side of the jejunum was then anastomosed with the gastric tube in an end-to-end manner using 3–0 PDS.

**Fig. 1:**
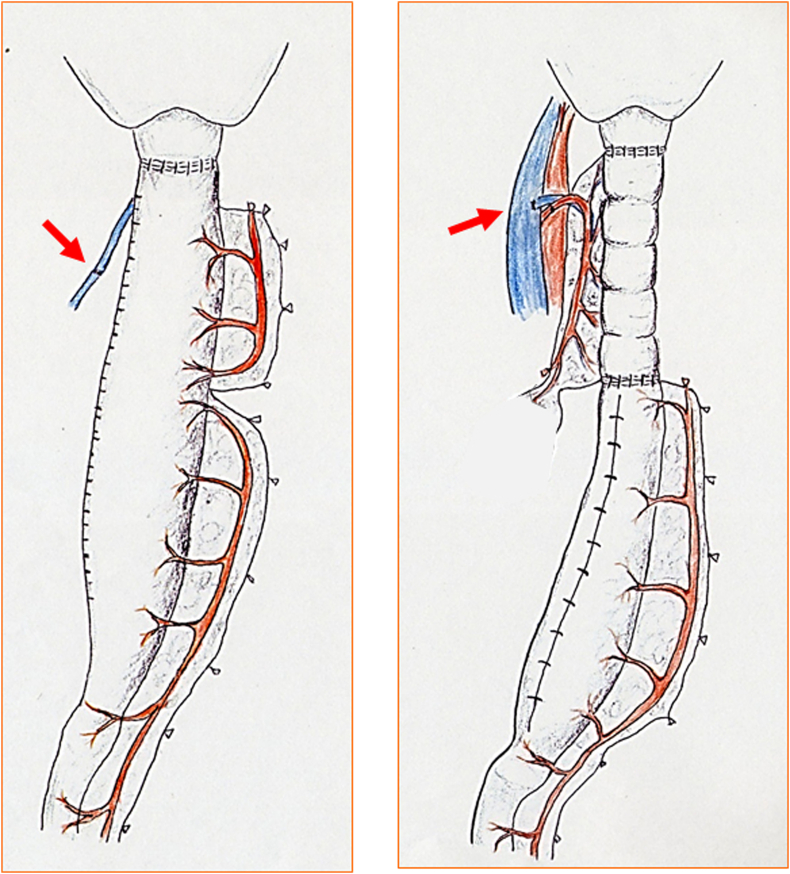
Operative schema. (a) Reconstruction with a gastric tube after total esophagectomy. In cases where venous microvascular anastomosis (super-drainage) was performed, the short gastric vein or posterior gastric vein was anastomosed to the anterior jugular vein or the internal or external jugular vein. Arterial anastomosis was sometimes added. (b) Reconstruction with a gastric tube and jejunal flap after total esophagectomy. Venous and arterial microvascular anastomosis was performed.

Microscopic venous anastomosis between the short gastric vein and cervical vein was performed when gastric tube reconstruction was principally performed; arterial anastomosis was added, if needed (Fig. 1). In patients with a FJF, vascular anastomosis (artery and vein) was performed. In most cases, the second jejunal artery and vein were anastomosed with the transverse cervical artery (in an end-to-end manner) and the internal jugular vein (in a side-to-end manner).

### 2.3 Statistical analysis

A p value of <0.05 was considered statistically significant. Analyses were performed using JMP statistical software version 14.0 (SAS Institute Inc., New York, NY, USA).

## 3 Results

### 3.1 Patient background characteristics

The median age was 65 years (range 36–80 years). The patients’ clinicopathological features are summarized in Table 1. Eight patients had synchronous esophageal cancer and head and neck cancer. Eighteen patients had a cervicothoracic tumor. Five patients with hypopharyngeal or laryngeal cancer were in the advanced clinical stage IVA. Twenty-two patients received preoperative chemotherapy or chemoradiotherapy. None of the patients received postoperative radiotherapy. Sixteen patients were reconstructed with only a gastric tube, while 10 patients were reconstructed with a gastric tube and FJF. Microvascular anastomoses using an artery and vein were performed in 10 patients who underwent reconstruction involving gastric pull-up combined with a FJF graft after TPLE. All patients underwent reconstruction via the mediastinal route. Venous anastomosis was performed for 13 of the 16 patients who underwent reconstruction with only a gastric tube.

**Table 1:** Clinicopathological features of the 26 patients who underwent thoracoscopic TPLE.

Case	Age	Sex	HNC	UICC stage of HNC	Location of main tumor	UICC stage of esophageal cancer	Reconstruction	Microvascular anastomosis	Curability	Preoperative therapy	Complications	Pneumonia	Leakage
1	36	M	None		Ce	0	Gastric tube	Vein	Curative	NAC	None	None	None
2	38	M	Hypopharyngeal cancer	II	Ce	III	Gastric tube	None	Curative	NAC	Present	None	None
3	50	M	None		Ce	0	Gastric tube	Artery/vein	Curative	NAC	None	None	None
4	56	M	None		Ce	III	Gastric tube + FJF	Artery/vein	Curative	NACRT	Present	None	Present
5	57	M	None		Ce	II	Gastric tube	Vein	Curative	NACRT	None	None	None
6	59	M	Laryngeal cancer	IVA	Mt	III	Gastric tube + FJF	Artery/vein	Curative	NACRT	None	None	None
7	62	M	None		Ce	II	Gastric tube	Vein	Non-curative	NAC	None	None	None
8	62	M	Hypopharyngeal cancer	IVA	Mt	IVA	Gastric tube + FJF	Artery/vein	Curative	NAC	None	None	None
9	63	M	Hypopharyngeal cancer	IVA	Ut	IVA	Gastric tube	Vein	Curative	NAC	None	None	None
10	64	M	None		Ut	I	Gastric tube	Vein	Non-curative	NACRT	None	None	None
11	64	M	Hypopharyngeal cancer	0	Ce	IVA	Gastric tube	Artery/vein	Curative	None	Present	None	None
12	66	F	None		Ce	II	Gastric tube + FJF	Artery/vein	Curative	NAC	None	None	None
13	66	M	None		Ce	I	Gastric tube + FJF	Artery/vein	Curative	NAC	None	None	None
14	66	M	None		Ce	III	Gastric tube + FJF	Artery/vein	Non-curative	dCRT	Present	None	None
15	67	M	None		Ce	IVA	Gastric tube + FJF	Artery/vein	Curative	NAC	None	None	None
16	69	M	Hypopharyngeal cancer	IVA	Mt	0	Gastric tube	Artery and vein	Curative	None	None	None	None
17	71	M	None		Ce	I	Gastric tube	None	Non-curative	NAC	None	None	None
18	73	M	Hypopharyngeal cancer	IVA	Mt	III	Gastric tube + FJF	Artery/vein	Curative	None	None	None	None
19	74	M	None		Ce	III	Gastric tube	Vein	Curative	None	None	None	None
20	74	M	None		Ce	I	Gastric tube	Vein	Curative	NACRT	None	None	None
21	74	M	None		Ut	IVA	Gastric tube	Vein	Non-curative	NAC	Present	Present	None
22	75	M	None		Ce	II	Gastric tube + FJF	Artery/vein	Non-curative	NAC	None	None	None
23	76	M	None		Ce	II	Gastric tube	None	Curative	NAC	Present	None	Present
24	77	M	None		Ce	III	Gastric tube + FJF	Artery/vein	Curative	NAC	Present	None	None
25	77	M	Hypopharyngeal cancer	III	Ce	II	Gastric tube	Vein	Curative	NAC	Present	None	None
26	80	M	None		Ut	II	Gastric tube	Vein	Non-Curative	NACRT	Present	None	None

TPLE: total pharyngolaryngoesophagectomy, HNC: head and neck cancer, FJF: free jejunal flap, NAC: neoadjuvant chemotherapy, NACRT: neoadjuvant chemoradiotherapy, dCRT: definitive chemoradiotherapy, UICC: Union for International Cancer Control.

### 3.2 Operative factors and complications

Table 2 summarizes the operative factors and complications. The median operation time was 760 min, median blood loss was 359 ml, and median ICU stay was 1.7 days. The total operation time differed in accordance with the type of microvascular anastomosis. The operation times for patients without microvascular anastomosis, with vein anastomosis, and with anastomosis of two vessels were 629 min, 764 min, and 787 min, respectively. Postoperative complications were assessed using the Clavien-Dindo (CD) grade [12]. Anastomotic leakage (CD grade ≥ 1) occurred in two patients; the leakage site was the pharyngogastric tube in one patient, and the pharyngojejunum in the other. Other complications (CD grade ≥ 1) were atrial fibrillation, and surgical site infection of the neck and lymphorrhea. There was no ischemia of the trachea or gastric tube. Only one patient died in hospital because of acute exacerbation of interstitial pneumonia.

**Table 2:** Operative factors and complications of thoracoscopic total pharyngolaryngoesophagectomy (*n* = 26).

Variable	Number (%)	Median (range)
Operation time (min)		760 (561–885)
Blood loss (ml)		359(250-1527)
ICU stay (days)		1.7 (1–5)
Anastomotic leakage	2 (7.7)	
Pneumonia	1 (3.8)	
Other complications	3 (11.5)	

## 4 Discussion

Previous study demonstrated the safe application of minimally invasive thoracoscopic surgery in TPLE for three patients with concomitant esophageal cancer and head and neck cancer, and showed that this method is less invasive than open surgery [8]. The case series showed that thoracoscopic TPLE and laparoscopic reconstruction with a gastric tube or FJF transfer was safe and effective in 26 patients.

Miyata et al. [13] reported that gastric pull-up combined with a FJF graft is a feasible reconstructive surgery after pharyngolaryngoesophagectomy via thoracotomy, with a reported operation time of 843 min and blood　loss　is　1300ml. In our case series, the median operation time was 760 min, median blood loss was 359 mL, and median ICU stay was 1.7 days. Thus, the thoracoscopic TPLE performed in our case series resulted in less blood loss and a shorter operation time than previously reported [13], which may be due to the thoracoscopic procedure and improvements in preoperative management. The operation time and blood loss were increased by the addition of a FJF or vascular anastomosis; however, the operative technique has also improved over time. Therefore, it is difficult to compare the present operative outcomes with previous reports [13].

The early postoperative complications after TPLE with gastric pull-up reconstruction in 208 patients were reported in detail by Shuangba et al. [14] In this previous study, the early postoperative thoracoabdominal complications comprised pneumonitis in 23 patients (11.1%), pleural effusion in 15 (7.2%), chylous fistula in four (1.9%), heart failure in four (1.9%), hemoperitoneum in two (1%), and burst abdomen in two (1%), while the in-hospital mortality rate was 0–16% [14]. Denewer et al. [15] reported a mortality rate of 10.6% and morbidity rate of 31.7%. The complications in the present case series were anastomotic leakage (7.7%), atrial fibrillation (3.8%), and surgical site infection of the neck and lymphorrhea (3.8%). There was no ischemia of the trachea or gastric tube. A previous study of TPLE via open thoracotomy reported an incidence of anastomotic leakage of 17%, and the occurrence of tip necrosis of the gastric tube [6]. Since the publication of this previous study [6], we have actively performed additional microvascular anastomoses or FJF interposition to prevent necrosis of the gastric tube and anastomotic leakage. Especially, FJF interposition is a useful technique for attaining tension-free anastomosis. In addition, we created a narrow gastric tube and performed microvascular venous anastomosis to prevent congestion [16,17]. Venous anastomosis (and sometimes arterial anastomosis) was performed in 13 of the 16 patients who underwent reconstruction with only a gastric tube (Table 1). None of these 13 patients had anastomotic leakage. Vascular anastomosis should be considered to prevent anastomotic leakage when using only a gastric tube. Our anastomotic methods and surgical procedures reduced the incidence of complications.

We performed TPLE via the most appropriate techniques in each case, including minimally invasive thoracoscopic and laparoscopic procedures, microvascular anastomosis, and FJF interposition. As a result, TPLE was routinely performed in many cases, demonstrating the feasibility and good outcome of this minimally invasive surgery and plastic surgery. The limitations of the present study were the single center design and small number of cases.

## 5 Conclusion

Thoracoscopic esophagectomy in the left hemi-prone position and laparoscopic reconstruction with a gastric tube in patients undergoing TPLE was safe and effective. The complications were improved via the development of various procedures. Further improvement is necessary before this thoracoscopic approach is established as a standard procedure for TPLE.

## Ethical approval

Hospital ethical committee permit and case registration(ID: 2019-212).

## Sources of funding

Drs. Yasue Kimura, Hiroshi Saeki,Qingjiang Hu, Yoko Zaitsu, Yuichi Hisamatsu, Mioko Matsuo, Sei Yoshida, Eiji Oki, Ryuji Yasumatsu, Hideaki Kadota, Masaki Mori have no conﬂicts of interest or ﬁnancial ties to disclose.Y

## Author contribution

Yasue Kimura: Conceptualization, Data curation, Formal analysis, Investigation, Methodology, Project administration, Resources, Software, Validation, Visualization, Writing - original draft, Writing - review & editing.Qingjiang Hu: Data curation, Investigation. Yuichi Hisamatsu: Data curation, Investigation. Mioko Matsuo: Writing - review & editing. Sei Yoshida: Writing - review & editing. Ryuji Yasumatsu:Writing - review & editing. Hideaki Kadota: Writing - review & editing. Hiroshi Saeki: Writing - review & editing. Eiji Oki: Writing - review & editing. Masaki Mori: Writing - review & editing.

## Registration of research studies

Unique Identifying number or registration ID: UMIN000040909

Hyperlink to your specific registration (must be publicly accessible and will be checked): https://upload.umin.ac.jp/cgi-open-bin/ctr_e/ctr_view.cgi?recptno=R000046702.

## Guarantor

Yasue Kimura

## Provenance and peer review

Not commissioned, externally peer-reviewed.

## Declaration of competing interest

The authors declare that they have no conflict of interest.
